# Thrombosis after Travel

**DOI:** 10.1371/journal.pmed.0030300

**Published:** 2006-08-22

**Authors:** Kenneth J Rothman

## Abstract

Rothman discusses a new study by Cannegieter and colleagues which looked at the interaction of lengthy travel with other risk factors for venous thromboembolism.

Traveling has always been a risky venture. In times gone by, the traveler left the security of home to face myriad dangers, including accidents, storms, infectious disease, malnutrition, and assault. Consider the choices faced by travelers from New York to San Francisco before the transcontinental railroad traversed North America in 1869. They had to choose between a harsh overland route by wagon train through forbidding territory, a sailing expedition around Cape Horn through some of the most treacherous waters on earth, or the most dangerous route, by sea to Panama, and then a trek by canoe, mule, and foot through mosquito-infested jungles in the hope of booking passage by ship from the Pacific side. By comparison, today's traveler would seem to have little to worry about.

## Risks of Modern Travel

But travel still entails some risk. True, vehicles are safer, hostilities are less common, and many other threats have been reduced. Nevertheless, it has become increasingly clear that modern travelers, especially long-haul airline passengers, face an increased risk of thromboembolism as a result of their travel [1–5]. The increase is modest, perhaps two to four times the baseline incidence of about one event in 1,000 person-years [6]. Age and other risk factors, however, greatly elevate the baseline risk that some travelers face. Given that one to six percent of long-haul air travelers may be arriving with a clot in their veins [4,7,8], most being asymptomatic [9], it appears that travel-related risk of thrombosis could be appreciable for those with high baseline risk.

What forms of travel, and what duration, affect the risk? Which determinants of thrombosis are susceptibility factors for travel-related risk? What interventions are effective? Some of these questions were addressed in a study by Cannegieter et al., appearing in *PLoS Medicine* [10]. The researchers analyzed data collected for a population-based case-control study of venous thrombosis conducted in the Netherlands [11]. Corroborating earlier work [1,2,4,5], they found a doubling in the eight-week risk of venous thromboembolism in travelers who journeyed four hours or longer. The increase was similar for travel by

Traveling has always been a risky venture.

air and for travel by car, bus, or train. The authors' main focus, however, was on the interaction of lengthy travel with other risk factors. For all forms of travel, but especially for air travel, they found that factor V Leiden thrombophilia, body mass index greater than 30, height greater than 1.9 meters, and use of oral contraceptives were strong susceptibility factors. In addition, height of less than 1.6 meters was also a susceptibility factor for air travel.

Epidemiologists have had a difficult time studying interactions because this objective usually requires substantially more data than estimating primary effects. When feasible, however, the estimation of interaction effects can elucidate causal mechanisms and even point the way toward targeted prevention. Thus, while all long-haul travelers are advised to wear loose clothing, move about periodically, and to exercise in place, tall or heavy people, those with factor V Leiden, or women taking oral contraceptives might additionally consider wearing graduated compression stockings or taking heparin prophylactically [9].

## Matching in Case-Control Studies

Cannegieter et al. employed an unusual study design, using individually matched controls who were the partners of cases. Contrary to intuition, matching in case-control studies does not control confounding, and ironically introduces bias toward the null when the matching factors are related to the study exposure. The bias arises because controls are intended to provide an estimate of the exposure distribution in the population from which the cases arise, whereas matching on factors related to exposure in selecting controls distorts the sampling. Consequently, matching controls to cases for correlates of exposure requires analytic methods to remove the selection bias [12]. In this study, however, the use of partners as controls provided a convenient sample of the source population for cases, and indirect control for various lifestyle factors that might be difficult to measure. The investigators removed the bias introduced by matching (manifest, for example, in the tendency for partners to travel together) with an appropriate analysis.

Using partners as controls resulted in most controls having the opposite sex as their matched case. Even so, Cannegieter et al. were able to control sex differences in the analysis. They apparently believed, however, that they could not use their matched case-control design to study the effect of oral contraceptives, because only women were users of oral contraceptives. Instead, they turned to a case-only analysis ([13]; see Glossary) just to evaluate the interaction between oral contraceptives and travel. Unfortunately, the case-only analysis assumes that travel is unrelated to oral contraceptive use, a tenuous assumption. Furthermore, it requires that interaction between travel and oral contraceptives be assessed as a departure from a multiplicative relation, which understates the extent of interaction. The assessment of biological interaction should be based upon measuring excesses above additive effects [12,14]. Indeed, if the relation is multiplicative, that in itself would indicate important interaction. Although the greater-than-multiplicative relation they found in this analysis suggests strong interaction, the case-only analysis was unnecessary. Despite the fact that oral-contraceptive use is confined to women, the authors could have studied its effect using their partner-matched case-control data. Think of being male as just a reason for a person to be unexposed. The matched analysis with control of sex would still yield valid results. They thus could have examined departures from an additive relation between oral contraceptives and travel-related risk, as they did for the other interactions that they studied, and without the need to assume that travel is unrelated to oral-contraceptive use.

## Persistence and “Dose-Response” Relation of Travel-Related Risk

Cannegieter et al. also displayed the number of cases by week since travel. This distribution, however, does not use information from controls, and could mislead regarding the persistence of travel-related risk. It would have been more useful to describe the relation between relative risk and time since travel directly, perhaps with spline regression. Similarly, it would have been instructive to plot another spline-smoothed curve showing the relative risk by duration of journey, to evaluate the “dose-response” pattern and particularly to see if there was an obvious time threshold. Future analyses of these data should elucidate these points.

**Glossary**

**Case-only analysis:** A type of case-control study in which the control series is replaced by information obtained from the case series.

**Multiplicative relation:** A relation between two risk factors in which the relative effects multiply. For example, if factor A doubles the risk and factor B triples the risk, their relation is multiplicative if those with both factors have six times the risk relative to those without either factor A or factor B.

**Spline regression:** A regression that estimates separate, but connected, regression line segments for different ranges of the predictor variable.

The current findings are nevertheless important both to scientists and long-haul travelers. The study also raises intriguing questions about the risk of sitting for several hours without moving, an activity—or should we say inactivity—considerably more common than travel.
